# OLDER ADULTS’ PERSPECTIVES ON USING AN AI-AIDED SMARTPHONE

**DOI:** 10.1093/geroni/igae098.4035

**Published:** 2024-12-31

**Authors:** Yi-Ling Hu, I-Chen Chen

**Affiliations:** Chag Gung University, Toayuang, Taoyuan, Taiwan (Republic of China); Da-Yeh University, Changhua, Changhua, Taiwan (Republic of China)

## Abstract

Falls and injurious falls are expected to increase with the aging population, causing significant distress. Developing an artificial intelligence (AI)-aided smartphone system to minimize home visits may reduce home visits and costs. Older adults’ perspectives on AI and related implementation tasks are crucial. Therefore, focus groups were conducted to explore older adults’ acceptance and concerns about using an AI-aided smartphone system. Community-dwelling older adults (over 65 years old) without cognitive impairment were recruited from local senior centers, hospitals, and the Department of Occupational Therapy at Chang Gung University (CGU), Taiwan. Two coders independently analyzed transcripts using a framework analysis approach. Data saturation was reached when no new ideas emerged in the final focus groups. The research team has conducted three focus groups with older adults (n=15). The main finding showed that older adults viewed AI-aided Smartphones as helpful but preferred traditional home programs with human contact; they were highly aware of falls but were concerned that new AI technology would steal their smartphone information. Other themes, such as fear of scams, lack of knowledge, and lack of age-friendly smartphone platforms, also emerged. The result of this study indicates implementing the AI-aided smartphone program for fall prevention is possible, but multiple barriers need to be removed for older adults. The finding may help health professionals aid traditional fall prevention programs with new technology and may advance program effectiveness.

